# Repurposing Antiepileptic Drugs for Cancer: A Promising Therapeutic Strategy

**DOI:** 10.3390/jcm14082673

**Published:** 2025-04-14

**Authors:** Noor Tarawneh, Shaymaa A. Hussein, Shtaywy Abdalla

**Affiliations:** 1 Department of Pharmacy, Faculty of Pharmacy, Al-Zaytoonah University of Jordan, Amman 11733, Jordan; n.tarawneh@zuj.edu.jo; 2 Department of Biological Sciences, School of Science, The University of Jordan, Amman 11942, Jordan; makishaymaa0077@gmail.com

**Keywords:** antiepileptic drugs, cancer, drug repurposing, lacosamide, topiramate, valproic acid

## Abstract

Epilepsy is a neurological disorder characterized by repeated convulsions. Antiepileptic drugs (AEDs) are the main course of therapy for epilepsy. These medications are given according to each patient’s personal medical history and the types of seizures they suffer. They have been employed for decades to manage epilepsy, thus delivering relief from seizures through numerous mechanisms of action. Aside from their anticonvulsant attributes, current evidence suggests that certain AEDs may display potential inhibitory effects against cancer invasion and metastasis. This review explored the complicated interactions between the modes of action of AEDs and the pathways causing cancer, and the potential impact of AEDs on the invasion and metastasis of various forms of cancer, while addressing their associated side effects. For example, valproic acid inhibits histone deacetylase, causing hyperacetylation of genes, especially those regulating cell cycle, culminating in cell cycle arrest. Topiramate inhibits carbonic anhydrase, thus disrupting the acidic microenvironment needed for cancer cells to thrive. Lacosamide increases the slow inactivation of the voltage gated Na^+^ channel, thus inhibiting the growth, proliferation, and metastasis of many cancers. Although drug development is a complex task due to regulatory, intellectual property, and economic challenges, researchers are exploring drug repurposing tactics to overcome these challenges and to find new therapeutic alternatives for diseases like cancer. Thus, drug repurposing is considered among the most effective ways to develop drug candidates using novel properties and therapeutic characteristics, and this review also discusses these issues.

## 1. Introduction

### 1.1. Background on Cancer

Cancer is a group of diseases that can develop anywhere in the body, causing chaos of cellular balance and threatening human life. At its core, cancer begins with the breakdown of the closely regulating mechanisms that control cell growth, division, and death. When normal cells are genetically altered, they lose their ability to conform to these regulating mechanisms, resulting in the uncontrolled proliferation and emergence of unusual masses of tissue identified as tumors [1].

Tumors can be classified as benign, in which the cells remain localized and relatively harmless, or malignant, where the cells possess the ability to invade neighboring tissues and spread to distant sites via a process called metastasis. This metastatic capacity is considered as the deadliest characteristic of cancer since it enables the cells to infiltrate organs and systems across the body, frequently with fatal consequences [2].

A wide range of circumstances can lead to the emergence of cancer, including genetic predisposition, environmental exposure, lifestyle choice, age, and infectious agents. Carcinogens, like tobacco smoke, ultraviolet (UV) radiation, and certain chemicals, can damage DNA and raise the chance of cancer initiation. In a comparable manner, infections with viruses, such as human papillomavirus, Epstein Barr virus, and Kaposi’s-associated virus, are involved in virus-associated cancers, and bacteria like Helicobacter pylori and other microorganisms may act as cofactors or as carcinogens for particular cancers [3].

Cancer treatment is multifaceted and often tailored to the specific type and stage of the disease. Healthcare providers utilize a variety of strategies to fight cancer. Some of these strategies are conventional like surgery, chemotherapy, radiation therapy, and immunotherapy, but some are advanced and innovative like targeted therapy, hormone therapy, stem cell transplantation, ablation therapy, nanomedicine, and other advanced therapies [4]. Treatment strategy depends on factors such as tumor features, patient decision, and overall health status.

Cancer remains a leading cause of death worldwide, motivating further efforts to produce innovative antineoplastic medications aimed at lowering mortality rates [5]. These efforts have culminated in the development of a wide spectrum of molecules with variable degrees of efficacy as anticancer agents. Drug development, however, is a lengthy and costly process that may take up to 15 years for a drug to reach the clinical use phase, demanding significant financial support from commercial and scientific institutions. Clinical investigations play a pivotal role in assessing the safety and efficacy of new medications in patients, yet the success rate of novel compounds in such studies remains insufficient, with less than 10% of tested drugs progressing to the clinical use phase [6].

### 1.2. Objectives of the Review

This review seeks to highlight the shared pathways and mechanisms between cancer and epilepsy, as well as to identify prospective treatment targets. This could include pathways involved in cell proliferation, apoptosis, angiogenesis, and DNA repair, among others that are dysregulated in both cancer and epilepsy. The review is focused on AEDs that have demonstrated promise in clinical trials or preclinical studies for repurposing to cancer treatment. Positive results in clinical trials indicated that some AEDs may effectively target cancer cells or impede tumor growth via mechanisms originally intended for epilepsy treatment. In this review, we systematically reviewed over 135 scientific articles that dealt with the concept of drug repurposing, its advantages, obstacles limiting repurposing, and the evidence supporting the use of antiepileptic drugs as cancer treatment. The findings of this review may contribute to expanding the evidence supporting drug repurposing (DR) as a viable strategy for advancing cancer treatment.

### 1.3. Cancer and Epilepsy

Epilepsy and cancer are two diverse disorders with significant morbidity and mortality. They may arise in the same person, presenting a complex clinical problem. Brain tumors are the most prevalent solid neoplasms diagnosed in patients with epilepsy [7]. In contrast, around 35–70% of patients with primary brain cancer will suffer from epilepsy at some point during their illness [8]. In brain tumor patients, there are two distinguished peaks in seizure occurrence: one after initial diagnosis of tumor and one during tumor [9]. Epilepsy has a peak incidence in early childhood, with a second peak occurring for those aged 60 years and older. Seizures triggered by recurrent tumors are more difficult to treat, significantly affecting the patient’s quality of life and potentially contributing to impaired function and cognitive ability [10]. Therefore, a comprehensive understanding of the mechanisms generating tumor-associated epilepsy as well as suitable therapeutic techniques is necessary.

Primary brain tumors and brain metastases are considered to account for 15–95% of epilepsy provoked by structural brain defects [11]. The conventional care strategy involves surgery, radiation therapy, and medications with AEDs [12,13]. Despite these treatment options, many patients with tumor-associated epilepsy continue to have disappointing results [14]. An emerging hypothesis associated some AEDs with metastatic cancer or with increased risk in some patients [15,16]. To successfully deal with the dual burden of epilepsy and cancer, translational and interdisciplinary strategies are required, with carful investigation of both direct and indirect impacts of AEDs on tumor biology [13]. This information can then be used to enhance care practices and to strengthen patient outcomes. The correlation between epilepsy and cancer represents complicated biological interactions that have considerable consequences for disease progression and treatment outcomes. Epileptogenesis, for example, is caused by many factors, among which is inflammation of neurons and glia cells [17]. Neuroinflammation manipulates pathways that lead to cancer through the release of cytokines and chemokines.

**Figure 1:**
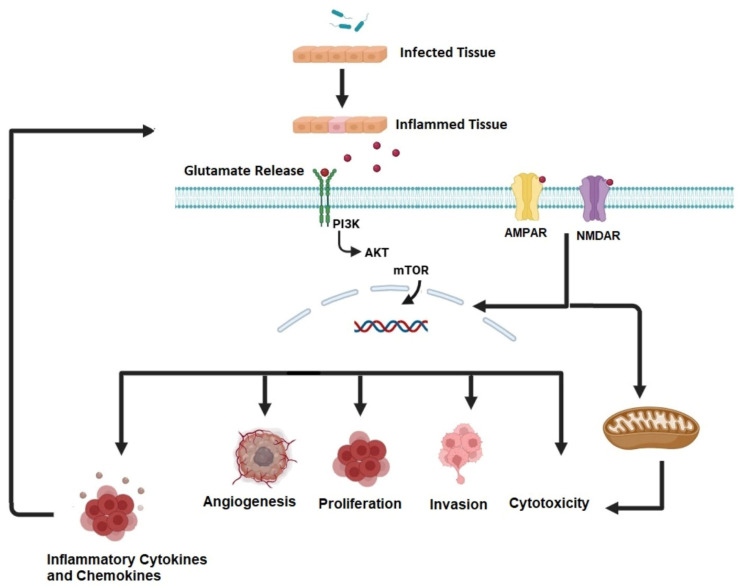
**Interrelations of inflammation and cancer.** Pathogen exposure triggers inflammation, causing cells to release glutamate. Glutamate interacts with receptors like tyrosine receptor kinases, AMPAR, and NMDAR, leading to cytotoxicity, invasion, proliferation, and the release of inflammatory cytokines and chemokines, which promote further glutamate release from inflamed tissues. Created using BioRender.com (accessed on 5 January 2025).

Inflammation and cancer are closely associated, with each possessing an impact on the progression of the other. Tumors can initiate inflammatory responses, whereas inflammation can stimulate tumor growth and metastasis [18]. Tumor cells play a vital part in regulating the expression of chemokines and cytokines (Figure 1), which are important for chemo attracting inflammatory cells and establishing a microenvironment favorable to tumor growth [19]. Simultaneously, glutamate release in the inflamed regions near tumors contributes to the formation of an inflammatory environment. This glutamate release activates many different cells, causing not only inflammation but also DNA oxidative damage and epigenetic and genetic mutations. These modifications dynamically regulate cellular signaling pathways involved in proliferation, survival, and invasion, enabling tumor progression [20,21].

In epilepsy, a dysregulation of specific glutamate receptor subclasses, notably ionotropic NMDA and AMPA receptors and metabotropic mGluR1-8 receptors, may simultaneously occur [22]. This dysregulation, combined with diminished GABAergic inhibition, causes neuronal hyperexcitability, which is the hallmark of epilepsy. The hyperactivation of glutamate receptors, which results in an imbalance between excitatory glutamatergic and inhibitory GABAergic neurotransmission, disrupts the equilibrium of neuronal activity. This elevated excitability exposes individuals to spontaneous seizures, representing the underlying pathophysiology of epilepsy [23].

**Figure 2:**
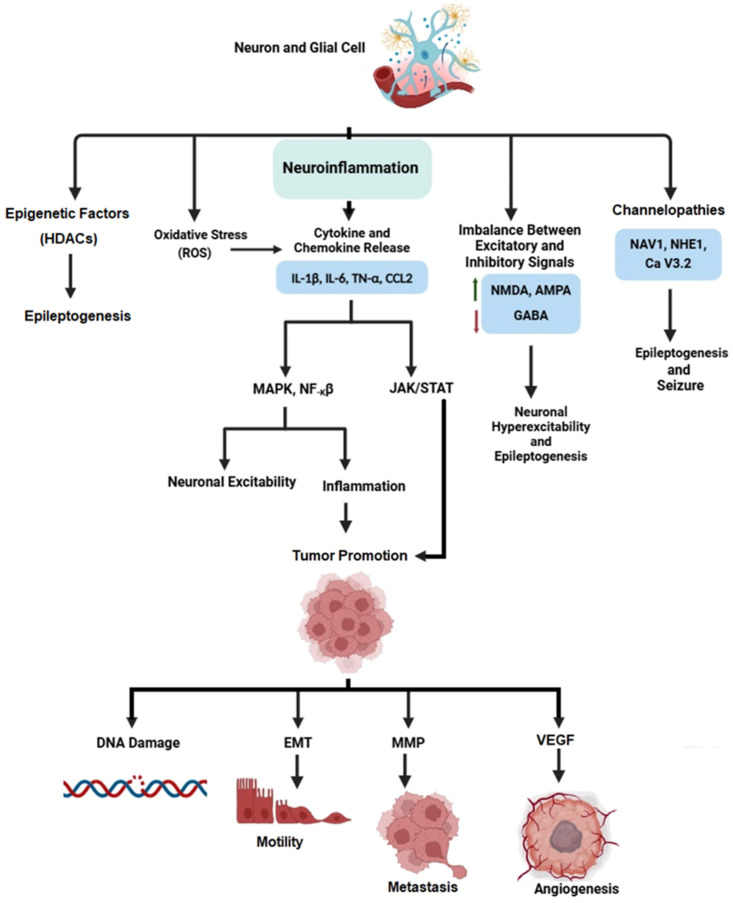
**Intricate interactions of neuronal inflammation, epilepsy, and cancer.** In neurons and glia cells, epigenetic factors, oxidative stress, inflammation, imbalances of excitatory and inhibitory signals, and channelopathies, all participate in epileptogenesis. Neuroinflammation, in particular, makes the connection between epilepsy and cancer through the release of cytokines and chemokines that activate inflammatory pathways such as JAK/STAT3, NF-κB, and MAPK, which enhance tumor promotion. Tumor promotion includes epithelial–mesenchymal transition (EMT) to facilitate metastasis, matrix metalloproteinases (MMPs) that facilitate migration, and growth factors like vascular endothelial growth factor (VEGF) that facilitates angiogenesis. Created using BioRender.com (accessed on 5 January 2025).

The interaction of these mechanisms illustrates the complex relation between inflammation, cancer, and epilepsy (Figure 2). Understanding these interrelated pathways holds promise for identifying novel treatment approaches for both cancer and epilepsy. Focusing on common pathways may result in synergistic effects in treating multiple illnesses, demonstrating the necessity of integration and understanding of shared pathways when managing diseases and developing drugs.

### 1.4. The Need for Drug Repurposing (DR)

To overcome such obstacles as tumor heterogeneity and drug resistance, numerous organizations are thoroughly examining DR. The approach of repurposing an existing drug involves identifying new therapeutic applications for a drug that has been previously approved or in clinical development for another purpose. This strategy may mitigate many of the difficulties associated with developing novel drugs from scratch [24]. Furthermore, DR can offer a lower-risk business model compared to traditional drug development by reducing some of the uncertainties and challenges associated with novel drug discovery. This is due to the fact that the safety and pharmacological profiles of repurposed drugs are already well-established, which decreases the likelihood of unexpected adverse effects or regulatory difficulties. However, despite these advantages, many repurposed drugs still fail in Phase II trials, which primarily assess safety, highlighting the ongoing challenges in the drug repurposing process. This and many other issues of DR have been discussed in significant detail in the review of Juárez-López and Schcolnik-Cabrera [6]. Overall, DR shows promise for solving unmet medical needs, accelerating the availability of treatments, and perhaps offering cost-effective solutions for both pharmaceutical companies and healthcare systems.

Historically, DR has been accidental or serendipitous, with many unplanned discoveries that stimulated the progress. The story of Viagra is one of the most famous examples of DR in modern medicine. In the late 1980s, researchers at Pfizer pharmaceutical company were working on developing a drug to treat angina pectoris, a condition characterized by chest pain due to restricted blood flow to the heart. The researchers were focusing on the compound sildenafil citrate, which they believed would dilate blood vessels and improve blood flow, particularly to the heart. The aim was to develop a drug that could reduce high blood pressure and alleviate symptoms of angina. During the clinical trials, participants (mostly males) began reporting an unusual side effect; they experienced improved erections. This was a surprising finding, as no one had predicted this effect. The increase in blood flow caused by sildenafil appeared to primarily target the corpora cavernosa, the sponge-like tissues in the penis, leading to more sustained and firmer erections [25].

Aspirin is another common example. This drug has gained popularity worldwide due to its multiple uses, such as relieving pain, reducing fever, and combating inflammation. In the 1950s, Lawrence Craven, a general practitioner from California, noticed that patients taking aspirin to prevent blood clots after surgery were experiencing lower rates of heart attacks. He began to recommend aspirin for heart and vascular health, although the scientific community was initially skeptical [26]. It took about 30–40 years before fully elucidating the COX inhibitory action of aspirin as it is known today.

However, recent advances have resulted in the establishment of systematic methods for identifying non-oncology drugs with potential applications in cancer therapy. These methodologies can be classified into computational and experimental approaches, which rely on high-throughput screening, bioinformatics tools, proteomic techniques, and cell-based phenotypic assays to identify promising candidates for repurposing [27,28].

Beta-blockers, for example, are known to treat diseases of the cardiovascular system, hyperthyroidism, and migraines, as well as glaucoma [29]. With the increasing popularity of drug repurposing, the anticancer effects of beta adreno-blockers are now being studied more extensively. The exact mechanism for their anti-non-small cell lung cancer (NSCLC) effects is still unknown, but many speculations exist. An experimental approach that studied the effects of beta blockers on the viability and cell colony formation of NSCLC showed that propranolol and betaxolol are the most effective in inhibiting lung cancer cell colony formation, at 90% of the EC_50_ value [30]. High-throughput drug screening and computational modeling identified propranolol as a potential inhibitor of angiogenesis, which is crucial for tumor growth and metastasis [31]. Proteomic approaches revealed that propranolol decreased expression of the pro-proliferative Ki-67 and pro-survival Bcl-2 markers and increased pro-apoptotic p53 expression in a patient with stage III breast cancer. Molecular analysis revealed that β-adrenergic receptor antagonism disrupted cell cycle progression (https://www.sciencedirect.com/topics/medicine-and-dentistry/cell-cycle-progression, accessed on 6 February 2025) and steady-state levels of cyclins (https://www.sciencedirect.com/topics/pharmacology-toxicology-and-pharmaceutical-science/cycline, accessed on 6 February 2025) [32]. Moreover, blockade of B1/B2-adrenoceptors was found to suppress pancreatic cancer cell proliferation and invasion by inducing apoptosis and by inhibiting the expression of NF-kB, AP-1 and CREB, as well as the expression of MMP-9, MMP-2, and VEGF target genes [33], all of which are pivotal for cancer management by propranolol. Metformin, a common medication for diabetes, has been repurposed as a potential treatment for cancer. Using bioinformatics tools and high-throughput screening, researchers have identified that metformin may target pathways associated with cancer metabolism. One of its primary mechanisms is activation of the AMP-activated protein kinase (AMPK) pathway, an energy sensor that inhibits processes like protein synthesis and cell growth, which are crucial for cancer cell proliferation. By inhibiting mitochondrial complex I, metformin reduces ATP production, leading to increased AMP levels and AMPK activation. This, in turn, suppresses the mammalian target of rapamycin (mTOR) pathway, which regulates cell growth and survival [34]. Metformin also influences the PI3K/AKT pathway, commonly dysregulated in cancer, by lowering insulin and insulin-like growth factor (IGF) levels, reducing cancer cell survival signals [35]. Furthermore, metformin inhibits hypoxia-inducible factor 1-alpha (HIF-1α), a transcription factor promoting angiogenesis and glycolysis under low oxygen conditions, thus hindering tumor growth [36]. Lastly, by affecting reactive oxygen species (ROS) production and regulating glycolysis and the pentose phosphate pathway (PPP), metformin starves cancer cells of the energy and biosynthetic materials needed for growth [37,38]. It inhibits respiratory complex I, leading to reduced ATP production, increased AMP/ATP ratio, and activation of AMPK signaling, followed by downregulation of mTOR, which inhibits PC cell proliferation [39]. These multifaceted actions make metformin a promising candidate for repurposing as a cancer therapeutic, especially in cancers with altered metabolic pathways.

Computational methods, such as gene expression data extraction and pathway analysis, have revealed metformin’s ability to inhibit the AMPK-mTOR pathway, which plays a significant role in cancer cell growth and survival [40]. In another study, cell-based phenotypic assays demonstrated that metformin inhibited the growth of several cancer types, including breast and colorectal cancers, by altering cancer cell metabolism and reducing insulin-like growth factor (IGF) levels [41,42].

DR, which investigates the possibility that existing medications have undiscovered anti-neoplastic effects, is a promising strategy for developing novel anticancer therapies. This strategy has financial advantages, since it allows for faster clinical trial initiation and may reduce the risks associated with “first-in-man” testing of completely novel drugs [43].

Overall, DR is a potential strategy for accelerating the development of innovative cancer therapies through employing current medications with known safety profiles. By systematically integrating data from various disciplines, including chemistry, pharmacology, and clinical research, DR holds the potential to uncover novel therapy options for cancer patients and improve outcomes in the fight against this devastating disease. Repurposing strategy necessitates the systematic assimilation of research data from various disciplines, including synthetic chemistry, bioinformatics and in silico modeling, systems pharmacological approaches, in vitro screens, clinical studies, and in vitro and in vivo functional assays [20].

### 1.5. A Practical Approach to DR

The conventional approach to drug research and development is a complex and resource-intensive process. DR based on useful strategies, like in silico screening and using the power of computer tools, examines large datasets that include patient data, disease targets, and medication information. In the in silico screening or molecular docking, a researcher first has to find, if possible, the receptor target molecule in the pathway leading to cancer growth and proliferation (e.g., PI3K), then to screen a compound or groups of compounds to find the best complementarity between any of these compounds (the ligands) and the receptor molecule like PI3K. Molecular docking can provide data to predict the strengths of binding energetics between the ligand and the binding site of the receptor molecule [44], presumably indicating the binding affinity of the ligand–receptor complex. The in silico screening uses a molecular approach that utilizes data from genomic, transcriptomics, and proteomics investigations on the drug targets and the structure of the molecule to accelerate DR. One such good example is the repurposing of the antibiotic gentamycin to treat Duchenne muscular dystrophy. This muscle dystrophy results from a premature stop codon that makes the muscle cells unable to produce dystrophin, a structural protein needed for muscle function. Gentamycin was found to allow translation through that stop codon and therefore restore functional dystrophin, allowing muscle cells to function better, although problems with the side effects of gentamycin are still to be dealt with [45]. Through the process of identifying probable medication interactions with emerging disease targets, researchers can better anticipate potential side effects, optimize therapeutic efficacy, and develop more personalized treatment strategies. Furthermore, existing information regarding a medication’s mode of action, safety profile, and side effects is a useful guide for repurposing initiatives. Researchers can find possible new applications for the medicine through comprehensive data analysis based on common biological pathways between the original and the new target diseases.

On the other hand, the phenotypic screening approach involves evaluating existing drugs against the disease of interest. Researchers are revealing the responses by observing the cell response to the drugs, allowing them to identify candidate drugs that show potential for treating the illness. As an illustration, researchers may use cell lines originating from a specific cancer type to test approved drugs. Drugs that inhibit cancer cell growth or induce cell death would be favored for further investigations. Using these diverse methodologies, researchers can uncover the latent potential of current medications for novel therapeutic uses. DR is a promising method for accelerating therapy development and eventually improving patient care [46,47].

### 1.6. Why Are Antiepileptic Drugs Repurposed for Cancer Treatment?

The story concerning the use of AEDs for cancer treatment is still developing. AEDs are typically administered to manage epileptic seizures. However, research revealed that they have effects beyond modulating electrical excitability; potentially their effects overlap with some cancer chemotherapeutic drugs at the cellular and molecular levels. For example, Aroosa et al. (2023) [20] provided an intriguing exploration of the potential for AEDs to be repurposed as cancer therapies. Traditionally, AEDs are used to control seizures by modulating electrical excitability in the brain, but the Aroosa study revealed that they may have broader effects, especially at the cellular and molecular levels. The research explored how AEDs might impact cancerous cells by influencing ion channels, which play roles in cell proliferation, migration, and survival, processes that are essential in both epilepsy and cancer. Certain AEDs exhibited anti-cancer properties by altering cellular signaling pathways, inhibiting cell proliferation, and inducing apoptosis in tumor cells. Furthermore, the overlap between AEDs and chemotherapy agents includes modulation of calcium channels, sodium channels, and neurotransmitter release, which may impact cancer cell metabolism and survival [20].

AEDs have also been investigated for treating neuropathic pain, migraine, and a variety of other neurological illnesses [48]. These numerous applications beyond epilepsy show both advantages and challenges when testing AEDs for cancer or other clinical settings. For example, AEDs have been compared to local anesthetics, which also inhibit cancer cell proliferation and, in animal models, slowed down tumor growth and metastasis [49], since these two classes of drugs share overlapping properties. These properties include blocking the initiation and conduction of nerve impulses [50] through blockade of sodium channels, which is critical for stabilizing neuronal membranes and controlling abnormal electrical discharges associated with seizures [51]. Both drug classes also can indirectly affect other ion channels, including the calcium and potassium channels, which play roles in maintaining neuronal stability, thus modulating pain perception [52].

## 2. Repurposed Antiepileptic Drugs with Anticancer Potential

### 2.1. Valproic Acid (VPA)

**Figure 3:**
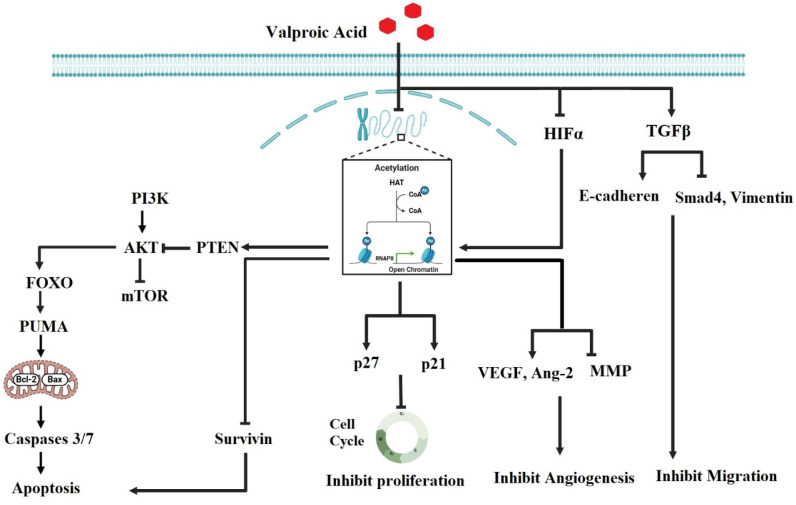
**Hypothetical mechanism of action of valproic acid.** Valproic acid exerts its effects by inhibiting HDAC activity and signaling pathways mediated by HIF-1α. The increased acetylation increases PTEN, which results in suppression of the PI3K-AKT pathway, ultimately leading to the inhibition of mTOR and associated proteins, including Bax, Bcl-2, survivin, and MMP. Consequently, this promotes the activation of caspase-3, caspase-7, p21, p27, VEGF, and Ang-2, resulting in apoptosis, reduced proliferation, and inhibition of angiogenesis. Additionally, valproic acid induces TGF-β, which suppresses Smad4 and vimentin while enhancing E-cadherin expression, thereby inhibiting cellular migration. This figure was created using BioRender.com (accessed on 5 January 2025).

VPA is a broad-spectrum medication primarily used to treat epilepsy and bipolar disorder. In light of several research findings, VPA exerts its therapeutic effects through multiple mechanisms, such as the modulation of neurotransmitter levels, but one of its significant actions is its role as a histone deacetylase (HDAC) inhibitor (Figure 3). By inhibiting HDAC, VPA increases the acetylation of histones, leading to a more relaxed chromatin structure and enhanced gene expression. This alteration in gene expression can have profound effects on neuronal plasticity and mood regulation, which are crucial for managing conditions like epilepsy and bipolar disorder. For example, VPA was found to regulate the expression of several neuroprotective genes, including brain-derived neurotrophic factor (BDNF) and B-cell lymphoma 2 (Bcl-2), which increases its therapeutic effects. BDNF is a neurotrophic factor that plays a crucial role in neuronal survival, growth, and plasticity, and VPA-induced increases in BDNF expression were found to be associated with enhanced neuronal resilience and function, potentially aiding in mood stabilization and reducing seizure susceptibility [53]. VPA also inhibited the expression of Bcl-2, an anti-apoptotic protein that prevent cell death in neurons, providing neuroprotective effects that may help stabilize neuronal circuits and minimize damage associated with epilepsy. By regulating these genes through its action as an HDAC inhibitor, VPA supports neuronal health and stability in both epilepsy and mood disorders, highlighting its wide therapeutic benefits [54].

Likewise, in the context of bipolar disorder, the modulation of gene expression may improve mood stabilization and mitigate the effects of mood swings. Furthermore, the HDAC inhibitory activity of VPA has been associated with anti-inflammatory effects, which may contribute to its therapeutic benefits in neuropsychiatric disorders. By influencing the epigenetic landscape, VPA not only addresses the immediate symptoms of epilepsy and mood disorders but also potentially alters the underlying pathological processes, offering a multifaceted approach to treatment [54,55,56]. The mechanism of action of VPA as an HDAC inhibitor has been used in a variety of medical problems, including epilepsy and cancer. VPA’s HDAC inhibitory action in epilepsy contributed to its antiepileptic effects by altering gene expression associated with neuronal excitability and seizure threshold [55]. Additionally, VPA raised interest in its potential to treat several types of cancer. For instance, researchers reported that VPA has the ability to inhibit cell growth and to induce apoptosis in the human colon carcinoma HT-29 [57] and hepatocellular carcinoma (HHC) PLC/PRF5 cell lines [58].

VPA’s property of hyperacetylation of histones caused the overexpression of genes involved in cell cycle regulation, such as cyclin-dependent kinase inhibitors (CDKIs) like p21 and p27. Increased expression of these CDKIs caused cell cycle arrest at various checkpoints, which inhibited cancer cell proliferation. VPA triggered apoptosis in cancer cells through multiple pathways. It promoted apoptosis by upregulating pro-apoptotic genes like Bax and PUMA and downregulating anti-apoptotic genes like Bcl-2 and Mc-1 [59].

VPA may disrupt the mechanisms that enable cancer cells to migrate and invade surrounding tissues. For instance, Zhang and collaborators found that VPA significantly inhibited the migration of human breast cancer (MDA-MB-231) cells. Mechanistic investigations revealed that VPA downregulated the expression of survivin, a protein that plays a crucial role in inhibiting apoptosis and promoting cell survival. Survivin is part of the inhibitor of apoptosis (IAP) family and helps cancer cells evade programmed cell death, thereby contributing to tumor growth and metastasis [60].

Survivin also influences cell division and has been implicated in various cellular processes, including cell migration. Its downregulation by VPA can lead to increased apoptosis and reduced viability of cancer cells, limiting their ability to migrate, which seems to be very much related to the function of aquaporins (AQPs) [61]. AQPs are water channel proteins that facilitate the movement of water and small solutes across cell membranes. They are known to play a significant role in cell migration by regulating cell volume and osmotic pressure. In the context of cancer, the expression of specific aquaporins, such as AQP1 and AQP3, has been associated with enhanced migration and invasion of tumor cells [62,63].

By downregulating survivin, VPA may likely affect the expression or activity of AQPs, potentially leading to reduced water influx and to changes in osmotic balance, which can inhibit cancer cell migration. This suggested effect of VPA on survivin and AQPs may contribute to limiting the spread of cancer to other parts of the body. Survivin inhibits apoptosis through multiple mechanisms, targeting key pathways of both intrinsic and extrinsic apoptosis. It interacts with caspase-9, an essential initiator in the intrinsic pathway, forming complexes with proteins like HBXIP to block apoptotic signaling. Additionally, survivin binds to pro-apoptotic factors such as SMAC/DIABLO, which are released from mitochondria during stress, thereby preventing these factors from neutralizing other inhibitors of apoptosis like XIAP. Although survivin does not directly bind effector caspases (e.g., caspase-3 or caspase-7), it works synergistically with other inhibitors of apoptosis to suppress their activation [64]. As a component of the chromosomal passenger complex, survivin supports cell division by ensuring proper chromosome segregation and cytokinesis, indirectly maintaining cell survival [65]. Furthermore, survivin modulates responses to extrinsic apoptotic stimuli, such as TRAIL and Fas signaling, by suppressing downstream caspase activation [66]. These mechanisms highlight survivin’s role in tumor cell survival and resistance to apoptosis, making it a critical target in cancer therapy.

Both survivin and AQPs, such as AQP1 and AQP5, are upregulated in response to hypoxic conditions in the tumor microenvironment, suggesting shared regulatory pathways, particularly via hypoxia-inducible factors (HIFs) [67,68]. Survivin regulation of migration and cell survival involves pathways like PI3K/AKT, which also influence AQP expression and activity [63,69]. Additionally, histone deacetylase inhibitors such as VPA, known to downregulate survivin, have been implicated in modulating AQPs in various cancer contexts [61]. Although direct evidence linking survivin to AQPs remains limited, these shared pathways and regulatory mechanisms suggest a possible interplay that needs further investigation.

VPA also modulated the expression of epithelial–mesenchymal transition (EMT) markers, a key process in cancer cell migration and invasion. However, its effects on EMT regulation differed across cancer types, with conflicting findings reported [70]. For example, Yang and coworkers (2022) observed that VPA inhibited EMT in glioma by increasing the expression of the epithelial marker E-cadherin while reducing the levels of the mesenchymal marker vimentin and Smad4 [71]. These changes suppressed the invasive potential of glioma cells, underscoring VPA’s antitumor properties. In contrast, Jahani et al. (2019) demonstrated that VPA treatment of gastric cancer cells reduced E-cadherin expression and increased vimentin expression, indicative of EMT induction [72].

The expression levels of E-cadherin and vimentin serve as key indicators of EMT status. E-cadherin, an epithelial marker, is essential for maintaining cell–cell adhesion in epithelial tissues. Reduced E-cadherin levels indicate the loss of epithelial characteristics, a hallmark of EMT that facilitates cancer cells in detaching from the primary tumor and migrating. Conversely, vimentin, a mesenchymal marker, is associated with increased cell mobility and invasiveness. Elevated vimentin levels signify a shift toward a mesenchymal phenotype, enhancing the migratory and invasive capabilities of cancer cells [73]. When VPA decreases E-cadherin and increases vimentin expression, it suggests an EMT-driven transition from a stationary epithelial state to a more migratory and aggressive mesenchymal state, potentially promoting cancer progression and metastasis. The contradictory findings regarding VPA’s role in EMT highlight its complex and context-dependent effects in cancer biology.

Additionally, VPA has been shown to inhibit angiogenesis, the process of forming new blood vessels that are essential for tumor growth and metastasis. VPA inhibited the expression of angiogenesis-related genes such as vascular endothelial growth factor (VEGF) and angiopoietin-2 (Ang-2), while also reducing the synthesis of pro-angiogenic factors like matrix metalloproteinases (MMPs). By targeting these pathways, VPA effectively restricted the blood supply to tumors, limiting their growth and ability to spread [74]. This anti-angiogenic effect contributed to its overall therapeutic potential in cancer treatment by impeding the vascular support that tumors require for continued expansion and metastasis.

VPA has been reported to influence several critical signaling pathways involved in cancer progression, including the PI3K/Akt/mTOR pathway, the Wnt/β-catenin pathway, and the NF-κB pathway. The inhibitory effect of VPA on the activation of the PI3K/Akt/mTOR pathway occurs through reducing the phosphorylation of Akt, leading to suppressed mTOR activity, decreased protein synthesis, and ultimately promoting apoptosis in cancer cells. This resulted in limited tumor growth and metastasis [75]. In addition, VPA modulated the Wnt/β-catenin pathway by inhibiting the translocation of β-catenin to the nucleus, preventing its accumulation in the nucleus where it activated target genes associated with cancer cell proliferation and invasion. By downregulating this pathway, VPA effectively reduced the expression of genes that promote tumor growth and metastasis [76]. Furthermore, VPA inhibited the NF-κB pathway by preventing the phosphorylation and degradation of the inhibitor of κB (IκB), which keeps NF-κB sequestered in the cytoplasm. This stabilization resulted in the inability of NF-κB to translocate to the nucleus and activate pro-survival and pro-inflammatory genes, leading to decreased cancer cell survival [77]. By modulating these pathways, VPA can effectively inhibit cancer cell proliferation, survival, and invasion, highlighting its therapeutic potential in repurposing for cancer treatment.

VPA can inhibit cancer cell proliferation, survival, and invasion. These findings highlight the therapeutic potential of repurposing VPA for cancer treatment. Current research suggests that VPA may be particularly useful when paired with established cancer therapies. Studies on the combination of VPA and temozolomide (TMZ), a chemotherapeutic medication, have shown favorable outcomes, as VPA improved TMZ anticancer activity in human glioma by increasing p53 activation and promoting the expression of its downstream proapoptotic protein, PUMA. PUMA (p53 upregulated modulator of apoptosis) is a proapoptotic protein that plays a vital role in mediating p53-dependent cell death, especially in response to cellular stress or DNA damage [78].

Li and colleagues reported that VPA can regulate specific cellular pathways when used along with gemcitabine, a standard treatment for pancreatic cancer [79]. Recent research suggested that it can enhance the effectiveness of known drugs such as cisplatin and doxorubicin (DOX), potentially offering improved outcomes for cancer patients [59,80]. For example, a recent study investigated the efficacy of combining VPA and cisplatin for treating triple-negative breast cancer (TNBC), an aggressive form with limited treatment options. The results showed that overexpression of aldehyde dehydrogenases (ALDH), the enzymes associated with drug resistance, may operate as one mechanism of cellular resistance to VPA in TNBC, and that the inhibition of ALDH effectively enhances the therapeutic efficacy of VPA-cisplatin drug combinations [81].

Intriguingly, Iannelli and his collaborators demonstrated the potential to repurpose treatment with two generic and safe drugs, VPA and simvastatin (SIM), in metastatic castration-resistant prostate cancer treatment through repressing a protein called YAP, which plays a crucial role in cancer stem cell function. By inhibiting YAP, VPA has the potential to eliminate these treatment-resistant cells and improve the overall treatment outcomes [82].

While pre-clinical and early clinical trials exploring VPA for cancer treatment are encouraging, it is important to remember that this area is still under active investigation. More research is needed to confirm VPA’s safety and efficacy in broader and more diverse patient populations. Nonetheless, the ongoing research on VPA as an anticancer agent is a testament to its exciting potential for the future of cancer treatment.

### 2.2. Topiramate (TPM)

A derivative of D-fructose sulfamate (2, 3:4, 5-bis-O-(1-methylethylidene)-β-d-fructopyranose sulfamate (https://www.sciencedirect.com/topics/chemistry/sulfamate, accessed on 5 January 2025) [83], TPM is a second-generation antiepileptic drug with a wide variety of pharmacological properties, including treatment of seizures, migraine headaches, and some mental problems [84]. The pharmacodynamics of TPM pertain to its actions on different neurotransmitter systems in the brain, especially boosting the activity of gamma-aminobutyric acid (GABA) and minimizing the activity of glutamate, the primary inhibitory and excitatory neurotransmitters, respectively [85].

**Figure 4:**
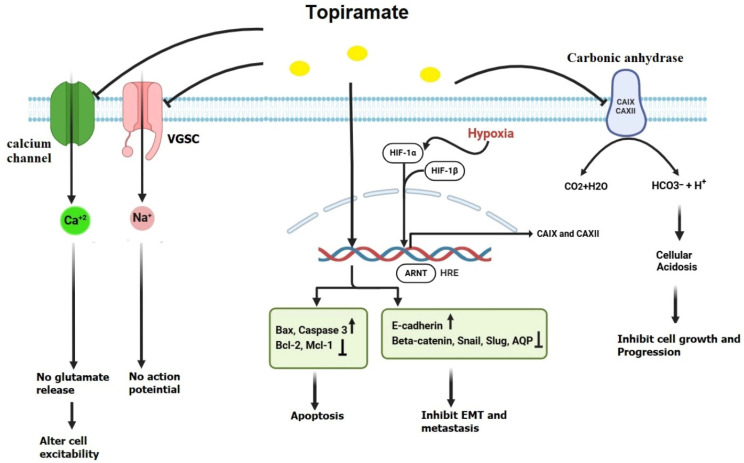
**Hypothetical diagram of the mechanism of action of topiramate (TPM).** Topiramate exerts its anticancer effects by targeting multiple pathways. It inhibits carbonic anhydrase isozymes (CA IX and CA XII), which are activated under hypoxic conditions, resulting in altered cellular pH that suppresses cancer cell growth and progression. TPM also regulates epithelial–mesenchymal transition (EMT) by downregulating key EMT regulators, including β-catenin, Snail, Slug, and AQPs, while upregulating E-cadherin expression, effectively inhibiting cancer metastasis. Additionally, TPM induces apoptosis by enhancing the expression of pro-apoptotic proteins such as Bax and caspase-3 and reducing the levels of anti-apoptotic proteins, including Bcl-2 and Mcl-1. Furthermore, TPM influences action potential and cellular excitability by inhibiting voltage-gated sodium channels (VGSCs) and calcium channels. This figure was created using BioRender.com (accessed on 5 January 2025).

TPM, like many other antiepileptic drugs, blocks voltage-gated sodium channels (Figure 4), which generate action potentials in neurons. By inhibiting these channels, TPM helps maintain the stability and polarization of neuronal membranes, thereby reducing the risk of abnormal electrical activity that can lead to seizures [51]. TPM can also influence calcium channels, which are involved in regulating glutamate release, and then alter neuronal excitability [86].

While TPM is well known for its antiepileptic and antimigraine properties, Xu et al. suggested that it may also have potential anticancer effects. However, this area of study is still in its early stages, and additional research is needed to fully understand the relationship between TPM and cancer inhibition [87].

Hypoxia is a critical feature of the tumor microenvironment, manifesting in various forms that range from moderate to severe, acute to chronic, and intermittent to persistent. These conditions drive diverse cellular responses, promoting aggressive tumor phenotypes [23,88,89]. At the molecular level, these hypoxic responses are primarily mediated by hypoxia-inducible factor (HIF), a key regulator of gene expression under low oxygen conditions [90]. One of HIF’s notable targets is the carbonic anhydrases (CAs), a family of cell-surface glycoproteins crucial for cellular adaptation to acidosis and cancer progression [88].

Among the CA isoforms, CA IX and CA XII are particularly significant in helping cancer cells thrive in the acidic microenvironment commonly associated with tumor growth. These enzymes regulate pH by catalyzing the reversible hydration of carbon dioxide into bicarbonate and protons, enabling cancer cells to maintain a neutral intracellular pH (pHi) despite extracellular acidosis. By buffering pHi and extruding protons, CA IX and CA XII support metabolic processes and survival mechanisms essential for tumor progression [91].

Inhibition of these enzymes disrupts the tumor’s ability to adapt to an acidic microenvironment, thereby preventing cancer progression. Tumor cells, characterized by elevated glycolytic activity even under aerobic conditions (the Warburg effect), produce excessive lactic acid and protons (H^+^), leading to an acidic extracellular environment [92]. To counteract this, cancer cells overexpress CA IX and CA XII, which catalyze the hydration of CO_2_ into bicarbonate (HCO_3_^−^) and H^+^, thereby maintaining an alkaline intracellular pH while acidifying the extracellular space. This acid–base regulation fosters cancer cell invasion and metastasis [91].

Targeting CA IX and CA XII impairs these adaptive mechanisms, resulting in intracellular acidosis that disrupts enzymatic functions and induces cell death. Concurrently, extracellular alkalization diminishes cancer cell migration and invasion. Beyond direct effects on cancer cells, CA inhibition mitigates the broader consequences of tumor acidosis, such as immune suppression, angiogenesis, and extracellular matrix degradation. By neutralizing extracellular acidity, CA inhibitors enhance immune function, inhibit blood vessel formation, and reduce protease activity critical for metastasis. This therapeutic strategy also enhances the efficacy of chemotherapy and radiotherapy, which are often less effective in acidic conditions. Thus, targeting CA IX and CA XII represents a promising approach to exploit cancer cells’ metabolic vulnerabilities, disrupting tumor adaptation to acidosis and inhibiting cancer progression [93]. Interestingly, one field of exploration focuses on TPM’s ability to disturb this favorable environment for tumors by inhibiting CA. In 2000, Dodgson and his colleagues evaluated the efficacy of TPM as an inhibitor of six CA isozymes (I–VI). The results showed that TPM inhibited all CA isozymes and revealed the capacity of TPM to inhibit CA II and CA IV more effectively than the other four CA isozymes [94].

TPM has shown promise in inhibiting cancer migration and invasion. Ma et al. studied the effect of TPM on lung cancer metastasis. The results showed that TPM downregulated AQP1 expression, inhibited CA activity, and prevented cancer metastasis by approximately 81.25% [95]. In 2018, Xu et al. investigated the anti-metastatic effects of TPM on ovarian cancer cells, particularly OVCAR5 and SKOV3 cell lines. They found that TPM decreased ovarian cancer cell adhesion and invasion, likely by influencing the expression of key EMT regulators. TPM was shown to upregulate E-cadherin expression, potentially maintaining the adhesive properties of the cancer cells and preventing epithelial–mesenchymal transition. Another important EMT regulator affected by TPM is ß-catenin, which is involved in both cell adhesion and the Wnt/ß-catenin signaling pathway. The activation of this pathway is known to promote cancer progression and metastasis. TPM appears to downregulate ß-catenin, inhibiting its activity and disrupting the pathway that is essential for maintaining the mesenchymal phenotype of the cells. This suggests that TPM’s inhibition of ß-catenin signaling may play a critical role in preventing ovarian cancer cell metastasis. In addition to E-cadherin and ß-catenin, TPM’s ability to downregulate vimentin expression implies that it can prevent the mesenchymal transition of cancer cells, reducing their motility and invasive capabilities. Moreover, TPM was found to affect two key transcription factors involved in EMT: Slug and Snail. Both transcription factors are known to repress E-cadherin expression and promote the mesenchymal phenotype. TPM likely downregulates the expression of Slug and Snail, preventing the loss of epithelial markers and inhibiting the transition to a more invasive mesenchymal state. By modulating these transcription factors, TPM reduces the migratory and invasive properties of ovarian cancer cells [87].

Several preclinical studies have yielded promising results regarding TPM’s potential anticancer effects. For example, several research studies have demonstrated that TPM inhibited the growth and proliferation of many malignancies both in vivo and in vitro. The study by Marathe et al. (2016) [96] investigated the anticancer effects of TPM on GBM, focusing on its ability to induce acute intracellular acidification. Using a mouse model, GBM tumors were generated by implanting U87 glioblastoma cells. The research applied a specialized magnetic resonance imaging (MRI) technique called amine/amide concentration-independent detection (AACID) chemical exchange saturation transfer (CEST) to monitor pH changes within the tumor microenvironment. The study revealed that a single dose of TPM (120 mg/kg) led to a significant acidification of tumor cells, as indicated by an increase in the AACID CEST effect. Importantly, this effect was tumor-specific, with no changes observed in the contralateral (non-tumor) brain tissue. These findings suggested that TPM altered the metabolic state of GBM cells, potentially disrupting their proliferation and survival mechanisms. The study highlighted TPM’s potential not only as a therapeutic agent but also as a tool for imaging-based tumor detection and treatment monitoring [96]. Chao et al. (2022) [97] investigated the anticancer potential of TPM in bladder cancer through in vitro and in vivo studies. The in vitro experiments demonstrated that TPM significantly inhibited the proliferation of bladder cancer cell lines 5637 and T24, an effect that was attributed to TPM-induced cell cycle arrest and apoptosis, as evidenced by increased expression of pro-apoptotic proteins Bax and caspase-3, alongside the downregulation of anti-apoptotic proteins survivin, Bcl-2, and Mcl-1. These findings were consistent across both cell lines and were further enhanced with increasing TPM concentrations. Additionally, TPM reduced the migration and invasion of bladder cancer cells, indicating its potential as an anti-metastatic agent. These effects were linked to the modulation of key molecular pathways, including PI3K/AKT/mTOR signaling. The in vivo studies using a bladder cancer model in BALB/C57 nude mice revealed that TPM treatment significantly reduced tumor size compared to controls, highlighting its tumor-suppressive efficacy. Importantly, TPM did not cause severe toxicity, suggesting its suitability as a safe therapeutic option. These findings underscore TPM’s potential as an adjunct or stand-alone treatment for bladder cancer, contributing to the growing evidence supporting its anticancer properties across various malignancies [97]. The study by Contreras-Zárate et al. (2023) [98] examined the use of TPM to prevent radiation-induced cytotoxic edema in preclinical models of breast cancer brain metastases (BCBM). Radiation therapy, a common treatment for BCBM, often leads to acute astrocytic swelling and edema due to the transient upregulation of AQP4 in astrocytes. This study demonstrated that short-term TPM administration effectively blocked AQP4 function, reducing astrocytic swelling, restoring trans-epithelial electrical resistance (TEER), and preventing brain water accumulation. Importantly, these effects occurred without promoting tumor growth or reducing the efficacy of radiation therapy. The findings highlight TPM’s potential as a temporary adjunct treatment to mitigate acute radiation-induced edema while complementing existing therapies targeting vasogenic edema, such as steroids or bevacizumab. This innovative approach leverages TPM’s safety profile and FDA approval, offering a clinically translatable solution to reduce treatment-related complications in BCBM patients [98].

### 2.3. Lacosamide (LCM)

LCM is a third-generation AED commonly used to treat epilepsy [99,100]. It has 100% bioavailability, and 95% of its metabolites are eliminated in urine [101].

LCM operates by modulating the voltage-gated sodium channels, enhancing the slow inactivation of these channels while having no effect on their fast inactivation. By enhancing slow inactivation, LCM prevents the channels from reopening and effectively terminates the action potential. This mechanism only impacts neurons that have been depolarized or activated for an extended length of time, such as those involved in epileptic activity. LCM also decreases the probability of seizures by reducing recurrent neuronal firing and stabilizing hyperexcitable neuronal membranes while retaining normal physiological function [102,103].

In addition to influencing sodium channels, LCM was found to inhibit collapsin response mediator protein 2 (CRMP-2), a protein involved in neuronal development and synaptic plasticity. This inhibition regulated neuronal excitability and reduced the abnormal development of neuronal connections in the brain, thereby contributing to its antiepileptic effects [103,104]. This regulation of CRMP-2 may have implications for other neurological conditions beyond epilepsy, such as schizophrenia, autism, alcohol dependence, depression, and bipolar disorders [105].

While LCM is primarily known for its management of epilepsy, emerging research suggests its potential as a candidate for cancer treatment. The precise mechanisms underlying LCM’s potential anticancer effects are still being elucidated; however, numerous hypotheses have been proposed based on its pharmacological properties [106].

**Figure 5:**
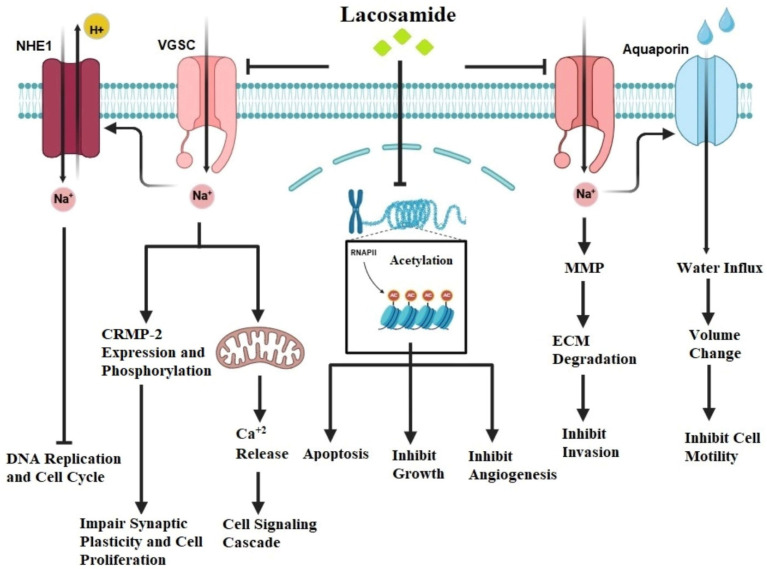
**Molecular mechanisms promoted by lacosamide (LCM).** LCM inhibits HDAC activity, resulting in suppressed cell growth, reduced angiogenesis, and induction of apoptosis. It also blocks VGSC, which physically interacts with the sodium-hydrogen exchanger (NHE-1) and aquaporins in specific membrane regions. By inhibiting VGSC activity, LCM reduces NHE-1 function, leading to localized peri-membrane intracellular acidification. This acidification inhibits DNA replication, disrupts the cell cycle, and decreases MMP activity, preventing the degradation of extracellular anchoring proteins and suppressing cell invasion. LCM also modulates local sodium concentration, reducing water influx through aquaporins, thereby altering cell volume and inhibiting motility. Furthermore, LCM suppresses CRMP-2 expression and phosphorylation, processes involved in cell proliferation. Additionally, it reduces intracellular calcium levels, inhibiting downstream signaling cascades via calcium-dependent kinases. This figure was created using BioRender.com (accessed on 5 January 2025).

Like in epilepsy, LCM’s major mode of action involves modulation of voltage-gated sodium channels in tumors (Figure 5). Voltage-gated sodium channels are found in cancers of the breast, colon, lung, prostate, cervix, ovary, lymphomas, and melanomas [107]. Aberrant expression and activity of sodium channels, often manifest as overexpression, increased activation, or dysregulated gating, have been found in cancer, contributing to cell proliferation, migration, and invasion [108,109].

LCM seemed to interfere with these processes by increasing the delayed inactivation of voltage-gated sodium channels, hence inhibiting cancer cell proliferation and metastasis [101]. Although no direct evidence exists confirming LCM’s modulation of sodium channels in cancer cells, delayed inactivation of these channels holds significant therapeutic potential by interfering with critical cellular processes driving tumor progression. Sodium influx through these channels influences intracellular sodium concentration, which regulates secondary sodium-dependent transporters such as sodium-hydrogen exchangers (NHEs). These transporters maintain a slightly alkaline intracellular pH essential for cancer cell survival, proliferation, and motility. Inhibition of sodium influx via delayed inactivation can cause intracellular acidification, thereby impairing DNA replication, cell cycle progression, and motility [110]. Conversely, persistent sodium channel activity drives membrane depolarization, which activates oncogenic pathways like PI3K/AKT and ERK that promote cancer cell growth and cancer cell survival, whereas delayed inactivation disrupts sodium-driven depolarization, attenuating these pathways and halting cancer progression [111]. Moreover, sodium channel activity is strongly connected to water movement through AQPs, a relationship critical for cancer cell migration and invasion. Sodium influx facilitates water entry via AQPs, enabling dynamic cell volume changes required for amoeboid migration, whereas reduced sodium influx due to delayed inactivation disrupts this sodium–water coupling, impairing cancer cell motility and invasiveness [112]. This interplay highlights the synergy between sodium channels and AQPs as promising therapeutic targets. Additionally, sodium channel activity regulates the expression and secretion of matrix metalloproteinases (MMPs), which degrade the extracellular matrix (ECM) and facilitate invasion, while delayed inactivation suppresses MMP transcription and secretion, thus reducing ECM degradation and metastatic potential. Furthermore, cancer cells often depend on sodium channel hyperactivity to maintain ionic homeostasis and metabolic activity under stress. Impaired sodium channel activity through delayed inactivation was found to disrupt calcium signaling, induce oxidative stress, and compromise cellular homeostasis, ultimately leading to apoptosis [113]. These multifaceted mechanisms underline the significance of targeting voltage-gated sodium channels in cancer treatment.

Cancer development and progression are linked to dysregulation of histone deacetylase activity. Granit and colleagues investigated the effects of LCM on histone acetylation in vitro. The cells of triple-negative breast cancer (MDA-MB-231) and human placental trophoblastic choriocarcinoma (BeWo) cells were treated with 20–80 μM LCM for 16 h. Western blot analysis was used to assess histone acetylation, and HDAC1 activity was also measured. At 20, 40, and 80 μM, LCM increased histone acetylation in BeWo cells by 1.7, 3.4, and 3.0-fold, respectively but the magnitude of change in histone acetylation in MDA-MB-231 cells was less. LCM may inhibit histone deacetylase in certain cell lines, increasing histone acetylation and affecting the expression of genes involved in cell proliferation, apoptosis, and tumor suppression, resulting in anticancer effects [114].

CRMP-2 is an important player in neuronal growth and axonal guidance [115]. Abnormal expression of CRMP-2, characterized by reduced expression and hyperphosphorylation, has been identified in a variety of malignancies including glioblastoma, breast, lung, and ovarian cancer [105,116,117,118]. For example, studies by Moutal et al. investigated the potential of lacosamide (LCM) to interfere with cancer cell proliferation, migration, and invasion by modulating collapsin response mediator protein 2 (CRMP-2), making it a viable target for cancer therapy. Moutal et al. studied CRMP-2 expression and phosphorylation in three human glioblastoma cell lines (GL15, A172, and U87) using cyclin-dependent kinase 5 (Cdk5) and glycogen synthase kinase 3 beta (GSK3β) to elucidate their roles in CRMP-2 regulation. To achieve this, they employed a combination of techniques, including Western blotting to assess CRMP-2 expression levels and phosphorylation status, utilizing specific antibodies against both CRMP-2 and its phosphorylated forms to quantify changes in response to treatment with LCM or specific inhibitors. Inhibitor studies were conducted using selective inhibitors of Cdk5 and GSK3β to understand their contributions to CRMP-2 modulation, enabling the evaluation of how changes in CRMP-2 phosphorylation affected glioblastoma cell proliferation and migration. Additionally, they performed cell proliferation assays along with immunofluorescence or immunocytochemistry techniques to visualize CRMP-2 localization within the cells, thus providing insights into its expression levels and changes in distribution that correlate with changes in cellular behavior. By integrating these methodologies, Moutal et al. observed that CRMP2 expression and phosphorylation regulated glioblastoma cell proliferation. The addition of CRMP2 phosphorylation inhibitor, (S)-lacosamide, an S-enantiomer of lacosamide, reduced cell proliferation and triggered apoptosis in all three glioblastoma cell lines examined in a concentration-dependent manner. In vivo models also demonstrated that (S)-lacosamide suppressed glioblastoma growth [105].

Rizzo et al. conducted an in vitro investigation to evaluate the efficacy of LCM on human glioma cell lines U87MG, SW1783, and T98G. The cell lines that were treated with 100–2400 μM LCM demonstrated a dose-dependent cytotoxic and anti-migratory effect. Furthermore, the exposure of human glioma cells to LCM modulated several microRNAs, particularly miR-195-5p, which appeared to affect cell cycle, and miR-107, which was shown to be implicated in the inhibition of cell migration [119].

Overall, while further research is needed to fully understand LCM’s anticancer mechanisms, its capacity to modulate sodium channels, histone deacetylase, CRMP-2, and various microRNAs suggests a promising role as a novel therapeutic agent in cancer treatment. Clinical studies are needed to assess its efficacy and safety in different cancer types and patient populations.

## 3. Challenges and Considerations

### 3.1. Dosage and Pharmacokinetics

Achieving therapeutic concentrations of antiepileptic drugs in cancer cells may require greater doses than those used in epilepsy treatment, thus raising the risk of side consequences [120]. Close monitoring of plasma drug levels and pharmacokinetic variables is required to optimize dosing policies and to reduce the toxicity in cancer patients [121].

AED doses are frequently modified based on variables such as age, gender, weight, kidney function, type and severity of seizures, and concurrent drugs [122]. Titration is necessary for many AEDs in order to progressively reach therapeutic levels and lower the possibility of side effects. For some AEDs, routine serum drug level monitoring may be required to guarantee therapeutic effectiveness and to prevent toxicity [123]. The bioavailability and absorption rates of AEDs can differ significantly, as some may be impacted by meal consumption. Their total efficacy is affected by how differently they diffuse in different bodily compartments.

VPA is usually titrated up to therapeutic levels (50–100 µg/mL) and may be taken once or twice a day. It has excellent oral absorption and strong plasma protein (i.e., albumin) binding (≥90%) [124]. VPA is extensively metabolized by the liver and has a half-life that varies from 10–20 h in adults to a significantly shorter duration (6–9 h) in children [125].

Starting with 15 or 25 mg per day, TPM is gradually raised up to 400 mg per day in split doses. It has a long half-life (∼24 h) in blood plasma with low level of binding to plasma proteins (≤20%). It is excreted primarily in urine as the parent compound (∼80%), and it additionally has excellent oral bioavailability [126].

LCM is initiated at 50–100 mg twice daily and can be increased up to 400–600 mg/day based on response and tolerability. It is minimally bound to plasma protein (˂15%) and its half-life is approximately 13 h. It has high oral bioavailability and undergoes minimal hepatic metabolism as well. Approximately 40% of the LCM dose is excreted as unchanged compound in urine [127]. A variety of drugs are metabolized by hepatic and extra-hepatic cytochrome P450 (CYP450) enzymes; drugs that inhibit or induce these enzymes may lead to increased or reduced efficacy or adverse events and toxicity, and may ultimately necessitate dose alterations in order to maintain appropriate therapeutic drug levels [128]. AEDs are primarily metabolized by the CYP450 system and other liver-based enzymes, which can lead to drug interactions. In patients with renal impairment, dose adjustments may be necessary, as renal excretion plays a crucial role in the elimination of many AEDs [124].

### 3.2. Drug Interactions

AEDs have the potential to interact with various cancer treatments, such as chemotherapy agents and targeted therapies, affecting their pharmacodynamics and pharmacokinetic characteristics [129]. Vigilant monitoring and dosage modifications are required in combination therapy patients to reduce the risk of adverse medication responses and assure therapeutic efficacy [130].

VPA interaction with cancer drugs can vary from beneficial, like enhancing the efficacy of chemotherapy agents, to potentially harmful, like increasing the toxicity of drugs. For example, Saha and his colleagues evaluated the mono- and combination-therapy effects of VPA and DOX in hepatocellular carcinoma cells. They discovered a specific and effective synergistic anti-proliferative effect of the VPA and DOX combination, particularly in HepG2 cells. However, this effect was not observed in MIHA cells, a normal hepatocyte cell line. The calculation of the coefficient of drug interaction confirmed the significant synergistic effect of the combination treatment. Concurrently, the synergistic apoptotic cell death caused by VPA and DOX combination treatment was confirmed by Hoechst nuclear staining and Western blot analysis of caspase-3 and poly ADP-ribose polymerase (PARP) activation. Co-treatment with VPA and DOX enhanced reactive oxygen species (ROS) generation and autophagy, which were clearly attenuated by the inhibitors of both ROS and autophagy. Furthermore, as an indication of the mechanism underlying the synergistic effect of the two compounds, the results revealed that DOX internalization, which was induced in the VPA and DOX combination-treated group, occurred through the caveolae-mediated endocytosis pathway [131]. Others investigated the antitumor effects of VPA in combination with cisplatin plus cetuximab (CX) doublet in head and neck squamous cell carcinoma models. The results showed that VPA enhanced DNA damage in combination treatment. This enhancement occurred through two mechanisms: First, it reduced the mRNA expression of ERCC Excision Repair 1, which is a critical player in DNA repair. Second, VPA increased the intracellular concentration of cisplatin by upregulating the transcription of the cisplatin influx channel copper transporter 1. Additionally, VPA downregulated ATP7B, an ATPase involved in cisplatin export [132].

VPA also induced a dose-dependent downregulation of EGFR expression and of MAPK and AKT downstream signaling pathways and prevented cisplatin- and/or CX-induced EGFR nuclear translocation, a well-known mechanism of resistance to chemotherapy. Indeed, VPA impaired the transcription of genes induced by non-canonical activity of nuclear EGFR, such as cyclin D1 and thymidylate synthase [132]. On the other hand, many drugs are metabolized by the hepatic cytochrome P450 isoenzyme system, and co-administration of AEDs and chemotherapeutic drugs can lead to clinically relevant interactions by induction or inhibition of enzymes in shared metabolic pathways. These interactions can cause insufficient control of tumors and seizures or lead to unforeseen toxicity. Inhibition of the metabolism of nitrosoureas or etoposide by VPA can lead to chemotherapeutic drugs toxicity. Poor seizure control may result from the combinations of VPA with methotrexate. The use of enzyme-inducing AEDs, such as phenytoin, carbamazepine, phenobarbital, and primidone, should be avoided in patients with cancer since these drugs can significantly induce cytochrome P450 enzymes, potentially reducing the plasma levels and therapeutic efficacy of chemotherapeutic agents such as tamoxifin, paclitaxel, and cyclophosphamide. This interaction may compromise cancer treatment outcomes while also increasing the risk of breakthrough seizures. Conversely, inhibition of drug metabolism by VPA can lead to the accumulation of chemotherapeutic drugs like cisplatin or etoposide, resulting in heightened toxicity.

Similarly, other AEDs like TPM and LCM can also interact with chemotherapy drugs. For instance, TPM induces the cytochrome P450 enzyme system, particularly CYP3A4 and CYP2C19, which can accelerate the metabolism of chemotherapy agents such as cyclophosphamide and paclitaxel, potentially reducing their efficacy [86,133]. On the other hand, LCM may have antagonistic effects when combined with cancer chemotherapy drugs, primarily due to pharmacodynamic and pharmacokinetic interactions that can alter treatment efficacy and toxicity. Pharmacodynamically, LCM’s neurological side effects, such as dizziness and ataxia, may intensify the neurotoxicity associated with certain chemotherapeutics, including platinum-based drugs (e.g., cisplatin, carboplatin) and taxanes (e.g., paclitaxel, docetaxel). This could result in increased neurotoxicity and potentially dose-limiting adverse effects [134,135,136]. Furthermore, while LCM is effective for seizure control, its interaction with chemotherapeutics that lower the seizure threshold, such as methotrexate, might compromise its effectiveness, potentially leading to breakthrough seizures. From a pharmacokinetic standpoint, LCM’s metabolism via CYP2C19 could compete with chemotherapy drugs that are metabolized through the same pathway, affecting their levels and efficacy. Additionally, LCM may alter the plasma protein binding of chemotherapeutics, influencing the free (active) concentrations of these drugs and potentially modifying both their therapeutic and toxic effects [130].

These interactions highlight the importance of careful monitoring and dose adjustments when using VPA or other AEDs in cancer therapy to ensure optimal therapeutic outcomes for both seizure management and cancer treatment [130].

## 4. Clinical Trials and Future Directions

Few in vitro and in vivo investigations reported the potential antitumor effects of approved AEDs. It is important to keep in mind that most research on AEDs and antitumor effects is in pre-clinical stages and has not reached the clinical phases, mostly for the considerations that we described in Section 1.5. For example, in 2004, Michaelis and colleagues investigated the anticancer effects of VPA both in vitro and in vivo. In vitro, therapeutically relevant concentrations of VPA (0.25 to 1 mM) inhibited proliferation, migration, and tube formation in human umbilical vein endothelial cells. At 1 mM, VPA reduced endothelial cell proliferation by 51 ± 5%, migration by 86 ± 11%, and tube formation by 82 ± 3%. These effects were associated with hyperacetylation of histone H4, indicative of histone deacetylase (HDAC) inhibition, and a reduction in endothelial nitric oxide synthase (eNOS) expression. The addition of the nitric oxide donor DETA NONOate [(Z)-1-[2-(2-aminoethyl)-N-(2-ammonioethyl)amino]diazen-1-ium-1,2-diolate] reversed the inhibition of endothelial cell tube formation caused by VPA. Notably, the VPA derivative 2-ethyl-4-methylpentanoic acid, which lacks HDAC-inhibitory activity, did not affect endothelial cell proliferation, tube formation, or eNOS expression.

In vivo, VPA inhibited angiogenesis in both the chicken chorioallantoic membrane assay and the Matrigel plug assay in mice. Additionally, embryos from VPA-treated mice exhibited disrupted blood vessel formation. These findings indicate that therapeutic plasma levels of VPA inhibit angiogenesis via a mechanism involving HDAC inhibition, leading to decreased eNOS expression [137,138].

The full scope of the anticancer mechanisms of VPA, TPM, and LCM remains unclear and is still under investigation. Moreover, their efficacy in in vivo studies involving humans are necessary to confirm these findings. The established uses and potential side effects of these AED medications need careful consideration before exploring their use in cancer treatment. The potential of AEDs as antitumor agents is a fascinating area of ongoing research. While VPA has taken the lead, other AEDs like TPM, LCM, lamotrigine, oxcarbazepine, and levetiracetam show promise. Further research is essential to determine their efficacy, safety, and ideal applications in cancer treatment. This holds the potential for novel therapeutic approaches and improved patient outcomes.

Several clinical trials are now being conducted in different laboratories to evaluate the possible therapeutic advantages of AEDs in cancer treatment [20]. These trials are intended to provide the mechanistic insights into the effect of AEDs within cancer cells, identify appropriate dosage schedules, and assess their efficacy when combined with conventional cancer treatments. Future investigations may explore the possibility of using AEDs as targeted therapy for certain cancer subtypes, following extensive molecular profiling and biomarker identification.

## 5. Conclusions

Antiepileptic drugs are a broad class of medications with different modes of action. Aside from their primary role in epilepsy management, certain AEDs have potential inhibitory effects on cancer invasion and metastasis, offering new opportunities for therapeutic investigation. This potential antitumor activity results from the fact that AEDs have modes of action that intersect with the mechanisms that promote cancer growth, proliferation, and metastasis. However, the clinical implications of these findings require further investigation, as well as careful consideration of associated side effects and individual patient profiles. Future research that clarifies the interaction between AEDs and cancer may find novel strategies for both neurological and oncological treatment paradigms.

## Data Availability

All data used to produce this work are available from the web and documented in the cited references. The corresponding author is ready to provide any document upon request.
